# Achieving integrated self-directed Cancer aftercare (ASICA) for melanoma: how a digital intervention to support total skin self-examination was used by people treated for cutaneous melanoma

**DOI:** 10.1186/s12885-021-08959-2

**Published:** 2021-11-13

**Authors:** Felicity Reilly, Lynda Contstable, William Brant, Kaz Rahman, Amer Durrani, Nigel Burrows, Charlotte Proby, Julia Allan, Marie Johnston, Derek Johnston, Fiona Walter, Peter Murchie

**Affiliations:** 1 Academic Primary Care Research Group, University of Aberdeen, Polwarth Building, Foresterhill, Aberdeen, AB25 2ZD UK; 2 Health Services Research Unit, University of Aberdeen, Health Sciences Building, Foresterhill, Aberdeen, AB25 2ZD UK; 3 NHS Grampian, Dr Gray’s Hospital, Elgin, IV30 1SN UK; 4 Aberdeen Royal Infirmary, NHS Grampian, Foresterhill, Aberdeen, AB25 2ZN UK; 5 Cambridge University Hospitals NHS Foundation Trust, Hills Road, Cambridge, CB2 0QQ UK; 6 University of Dundee, Division of Cancer Research, James Arrott Drive, Ninewells Hospital and Medical School, Dundee, DD1 9SY UK; 7 Health Psychology Group, University of Aberdeen, Health Sciences Building, Foresterhill, Aberdeen, AB25 2ZD UK; 8 Wolfson Institute of Preventive Medicine and Institute of Population Health Sciences, Barts and The London School of Medicine and Dentistry, Queen Mary University of London, London, UK

**Keywords:** Primary care, Melanoma, Cancer, Randomised Controlled Trial, Survivorship, Self-directed care, e-health

## Abstract

**Background:**

Melanoma incidence has quadrupled since 1970 and melanoma is now the second most common cancer in individuals under 50. Targeted immunotherapies for melanoma now potentially enable long-term remission even in advanced melanoma, but these melanoma survivors require ongoing surveillance, with implications for NHS resources and significant social and psychological consequences for patients. Total skin self-examination (TSSE) can detect recurrence earlier and improve clinical outcomes but is underperformed in the UK. To support survivors, the Achieving Self-directed Integrated Cancer Aftercare (ASICA) intervention was developed to prompt and improve TSSE performance, with subsequent reporting of concerns and submission of skin photos to a Dermatology Nurse Practitioner (DNP). ASICA was delivered as a randomized pilot trial.

**Methods:**

This paper reports on process evaluation. Data on participants’ demographics and the concerns they reported during the trial were tabulated and displayed using Microsoft Excel and SPSS. We explored which participants used ASICA, and how frequently, to report any skin concerns. We also determined how the interactions had worked in terms of quality of skin photographs submitted, clinical assessments made by the DNP, and the assessments and decisions made for each concern. Finally, we explored significant events occurring during the trial. Data on participants’ demographics and the concerns they reported during the trial were tabulated and displayed using SPSS. A semi-structured interview was undertaken with the DNP to gain perspective on the range of concerns presented and how they were resolved.

**Results:**

Of 121 recruited melanoma patients receiving ASICA for 12 months, 69 participants submitted a total of 123 reports detailing 189 separate skin-related concerns and including 188 skin photographs. Where participants fully complied with follow-up by the DNP, concerns were usually resolved remotely, but 19 (10.1%) were seen at a secondary care clinic and 14 (7.4%) referred to their GP. 49 (25.9%) of concerns were not completely resolved due to partial non-compliance with DNP follow-up.

**Conclusion:**

Melanoma patients randomized to the ASICA intervention were able to report skin-related concerns that could be resolved remotely through interaction with a DNP. Feasibility issues highlighted by ASICA will support further development and optimization of this digital tool.

**Trial registration:**

Clinical Trials.gov, NCT03328247. Registered on 1 November 2017

## Background

Melanoma is the fifth most common cancer in the UK, with over 16,000 diagnoses annually, and accounts for 1% of all cancer deaths [1, 2]. Although melanoma can be fatal, it has a high relative survival rate at five years, over 90% in the UK [2, 3]. However, those treated for melanoma are at risk of recurrence and the development of further primary melanomas in up to 8% of those initially diagnosed [4, 5]. Melanoma follow-up and ongoing surveillance of those treated for melanoma has an important role in detecting recurrence and new primaries at the earliest stage, when prompt treatment may improve outcomes [6]. Traditional structured follow-up, necessitating regular visits to a hospital specialist is increasingly costly and burdensome for patients and the NHS and the overall costs of providing NHS care to patients with skin cancer, in England only, has been calculated at £190.5 million in 2020 [2, 7].

As an adjunct to structured follow-up, most international consensus guidelines on melanoma management, including those of the British Association of Dermatologists and the Scottish Intercollegiate Guideline Network, recommend that patients should perform regular total self-skin examination (TSSE) in the intervals between hospital appointments to aid early detection [8–12]. This may prove instrumental in improving clinical outcomes with studies showing 62% of melanomas being first identified by patients themselves [13, 14]. Although widely recommended, the rate of skin checking by melanoma survivors is only similar to the general population [15]. Encouraginly evidence of how TSSE can be promoted and sustained is growing [16].

Digital technology develops apace and is recognized as a potential solution to a number of healthcare challenges, especially in rural areas where easy access to healthcare is often geographically limited [17–19]. Qualitative interviews with melanoma survivors have supported the view that, with appropriate training and design, smartphone-based app (application) technology is an acceptable way to promote and support TSSE [20].

**Fig. 1:**
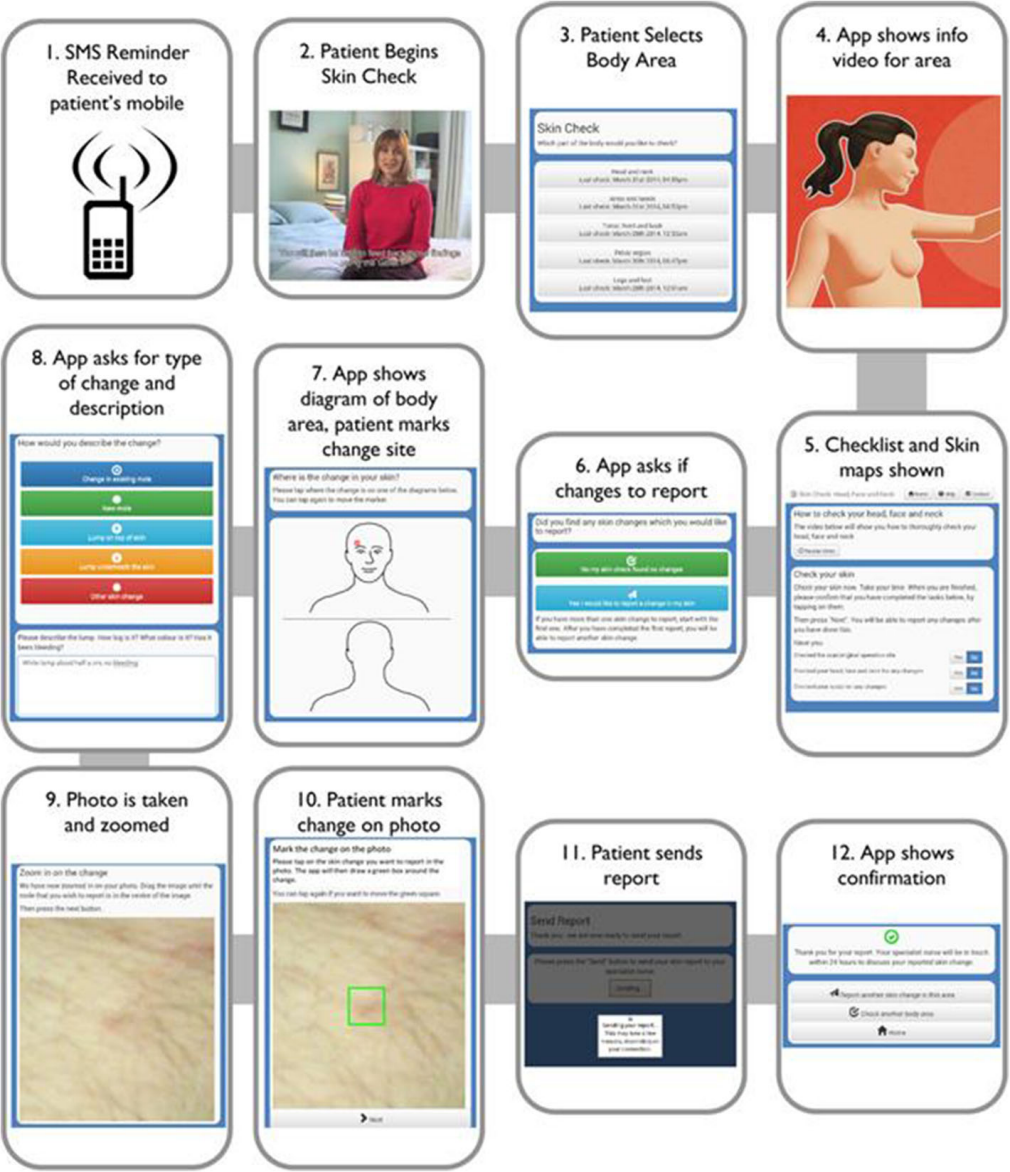
Schematic diagram representing the function of the ASICA intervention

The Achieving Self-directed Integrated Cancer Aftercare (ASICA) app was developed to prompt, support, record and respond to TSSE reports by patients who have completed primary treatment for melanoma. ASICA was an iteratively developed evidenced-based app intervention to support and improve TSSE adherence and practice, using tablet-based technology [21]. The app, hosted on Android tablets used animated instructional videos and monthly prompts to users to support TSSE. The app’s features included an individualised digital skin map and the facility to send electronic reports of any skin concerns, including photographs, to a remote Dermatology Nurse Practitioner (DNP), a specialist nurse with additional training and experience in clinical dermatology. (Fig. 1). Following development and feasibility testing, ASICA has undergone a feasibility randomised controlled trial (RCT) with a nested qualitative component to gather data on the experiences of users and intermediate clinical outcomes (to be reported elsewhere) [22]. The ASICA clinical trial aimed to improve clinical outcomes of melanoma recurrence and reduce the burden on patients and health services [22]. If the trial, completed in April 2020 (to be reported elsewhere), shows positive outcomes, the ASICA app may become a useful tool in melanoma aftercare within the NHS, particularly in rural areas.

### Aims

This paper reports on the clinical activity generated by the ASICA intervention. Firstly, we explored which participants had used ASICA. Secondly, we explored the frequency with which the participants who used ASICA reported concerns and the number of concerns that they submitted for consideration by the dermatology nurse practitioner (DNP). Thirdly, we explored the quality of skin photographs submitted, the clinical assessments made by the DNP, and the assessments made for each concern. Fourthly, we present the data on significant events occurring during the trial and the outcome of instances when users were referred to their GP or seen face-to-face in secondary care.

## Methods

The full methodology, including flow chart, is described in the published protocol [22]. This report focuses on the clinical activity generated by participants using the ASCIA intervention as part of a randomized controlled trial reported elsewhere. The full trial was registered at Clinical Trials.gov, NCT03328247 on 01/11/2017 (https://clinicaltrials.gov/ct2/show/NCT03328247?term=ASICA&rank=1).

Adults (over the age of 18) who had completed treatment within the previous 60 months for a stage 0-2C primary cutaneous melanoma were invited to participate in the ASICA feasibility RCT. Participants required wireless connectivity at home with sufficient capability to transmit images of skin concerns taken using the Samsung Galaxy 7″ tablets in-built camera. Patients were excluded if they had stage 3 or 4 melanoma, had a recurrence of melanoma within the last 60 months, were not able to consent to participate or complete questionnaires, or were blind or visually impaired.

ASICA was an open multi-centre two arm feasibility RCT which recruited 241 participants from two UK NHS secondary care sites (NHS Grampian and Cambridge University Hospitals NHS Foundation Trust). Ethical approval was given by the National Research Ethics Service (NRES) Grampian Ethics committee on 28th April 2017(Reference Number 17/NS/0040), and all participants gave written informed consent. No participant received any remuneration to take part.

**Fig. 2:**
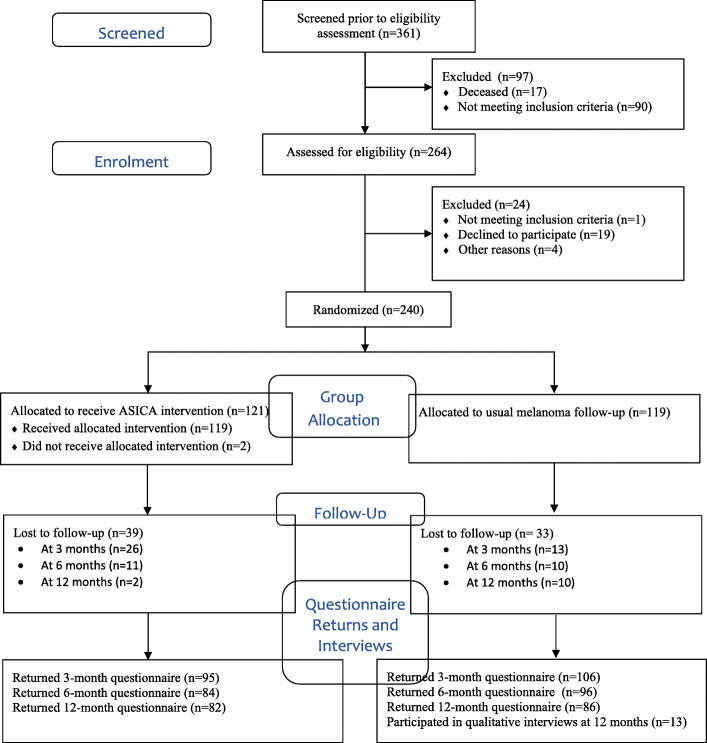
Modified CONSORT diagram showing patient flow in the ASICA study

Figure 2 is a modified CONSORT diagram showing the flow of participants into the intervention and control groups. Participants were randomized to the ASICA intervention plus standard care, or standard care alone in a 1:1 ratio, minimized on gender and centre, using a validated remote computer-automated randomization system hosted at the Centre for Healthcare Randomized Trials (CHaRT) in Aberdeen, UK. Blinding was not possible due to the requirement to operate the ASICA app (or not). This report focusses only on those participants randomized to the ASICA app, further information on participants in both groups will be presented elsewhere.

**Fig. 3:**
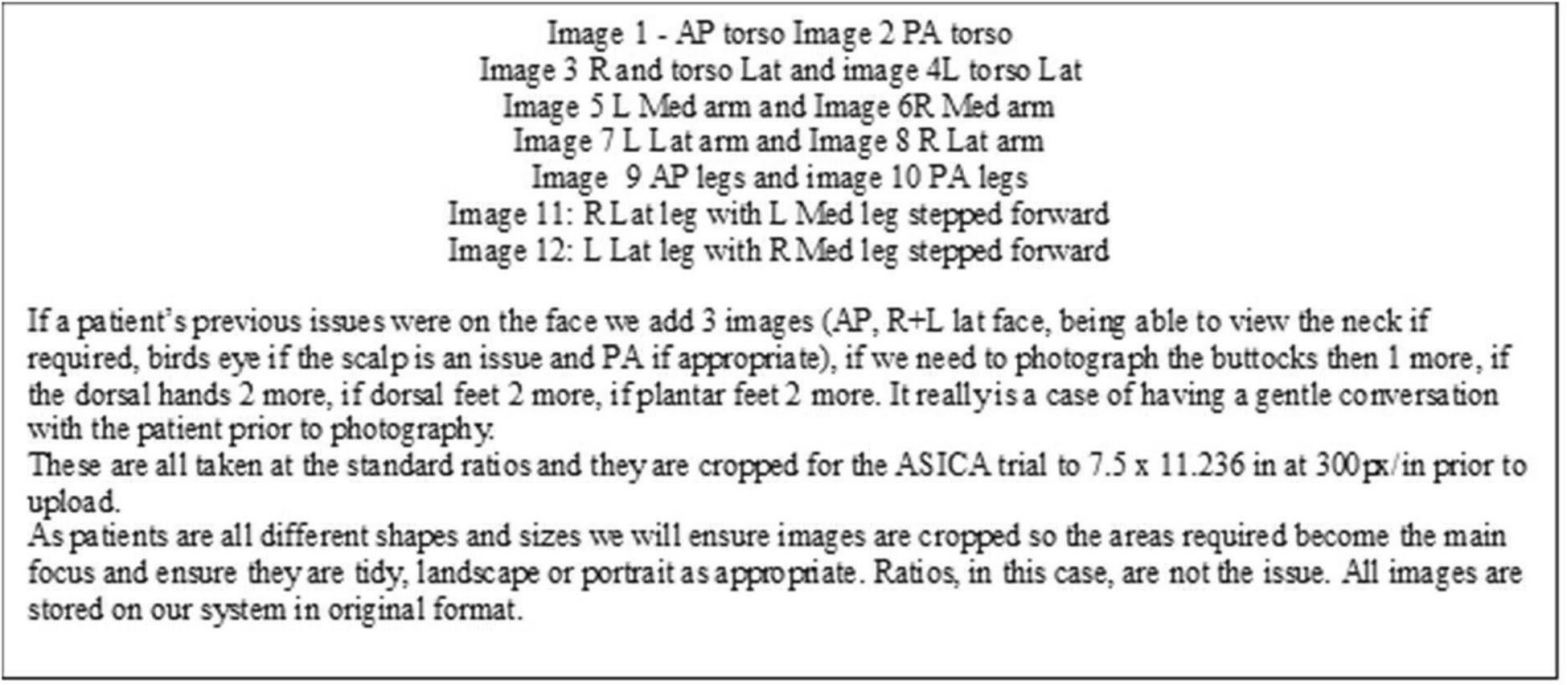
Standard skin-mapping protocol for ASICA digital skin maps

Participants randomised to the ASICA intervention were invited to attend the local medical photography suite to have a standard set of full body digital skin photographs taken. At their attendance it was explained that the intention was to take a series of digital images of their whole body to form a digital skin map to which they would be able to refer during the trial. Patient were then asked to provide written consent before the medical photographer took a set of 12 standard body map images using a digital camera (Fig. 3). These images were then used to create individual skin-maps which were uploaded to a secure server and individuals could refer to their own skin maps via the internet at any time during the trial. The ASICA intervention group received a Samsung Galaxy 7″ tablet preloaded with the ASICA app and received comprehensive training to use the app (in person, group and written instructions). The ASICA app included an instructional video on how to sequentially conduct a TSSE. Individuals could also use their Samsung Galaxy 7″ tablet to view their own individual digital skin map at any time. The device also included a digital camera and the app included a video which instructed participants how to take photographs of skin lesions or other concerns that they had. Finally, the app had a structured electronic TSSE report form which was used to send a report, including attached photographs, of each individual TSSE direct to the DNP for assessment and action as appropriate. Since participants all had experience of receiving melanoma follow-up examination no specific directions were given or restrictions made on the nature of skin concerns that they should report. No restriction was placed on submitting concerns from intimate body areas. The DNP recorded details of their assessment of the concern, including skin photographs, and their diagnosis and clinical decision on an electronic clinical portal which were revised and improved during the study.

The ASICA intervention group received monthly prompts (phone, email, text or mail) to conduct a TSSE and to take a photograph of any concerning skin lesions. The reports (and images) were uploaded to the hosted secure server, alerting the clinical nurse specialist (DNP) to review and follow up.

All participants continued with standard care and attended their usual structured melanoma follow up as per local guidelines. All participants also completed questionnaires (postal or web based, depending on preference) at 3, 6 and 12 months after randomization, with a further postal reminder if no response after 3 weeks. A clinical review of medical notes of all participants was also undertaken 12 months after randomization to collect any relevant pathology data. Additionally, a purposive sample of members of the intervention group were recruited to participate in telephone interviews with FR after 12-months. FR also interviewed the study DNP after 12 months.

When a participant reported a skin concern using the ASICA app (see Fig. 1), an automated alert was triggered and sent to the DNP for review. The DNP consulted with the participants directly by telephone and recorded their concerns. Data collected included:

- Area of body that concern related to
- The sharpness and focus of the skin photographs submitted and if further skin photographs were required
- DNP assessment of concern
- Further action recommended

Participant demographics and data recorded from the reports of concerns were collated, organized and tabulated using SPSS [23]. .The data were then cross tabulated by demographic group to determine potential differences and discrepancies across gender, age, study centre, and the deprivation and rurality categories linked to individual participants’ home address [24–27].

## Results

### Who submitted concerns?

In this paper we report data from the intervention group of the ASICA study focusing on those who notified concerns about their skin to the study DNP during the study year. Full details of recruitment and retention will be reported elsewhere along with the main trial results. To note, however, of 259 white Caucasian individuals of Scots, English, Irish and Eastern European origin across both sites who had been screened as eligible to participate and were invited to take part in the ASICA trial, 240 (92.7%) consented and 19 (7.3%) declined. Table 1 shows key demographics and the site of primary melanoma for all 121 members of the intervention group and separately those 69 members who submitted concerns to the DNP using the ASICA app during their 12 months of receiving the ASICA intervention. Compared to the intervention group overall, those who submitted concerns were younger and more likely to live rurally. Notably, overall six intervention group participants resided in the most deprived Scottish quintile, and only one of these six submitted a concern with their skin during the study year.. Submitters from the Grampian site numbered 49 (68%) with 20 (32%) from the Cambridge site. There were slightly more females than males submitters (55.1% to 44.9) and their mean age was 57.5 years (SD 13.6 years), but participants submitters concerns had a wide range of ages, both younger and older patients. Overall 6 (8.7%) submitters resided in the most-deprived five deciles compared to 15 (22%) coming from the single most affluent decile. [Scottish Government 2020; UK Government 2019] Over half of submitters were rural-dwellers (*n* = 38 (55.1%), with a similar proportion living rurally at each site, 28 (51.9%) from Grampian and 10 (50.0%) from Cambridge.

**Table 1:** Demographics of individuals who submitted concerns using ASICA (*n* = 69)

	Intervention Group (n%)	Submitted a concern (n%)	Grampian n(%)	Cambridge n(%)
**Participants**	121 (100)	69 (100)	49 (62)	20 (38)
**Gender**	**n(%)**	**n (%)**	**n (%)**	**n (%)**
**Male**	55 (45.5)	31 (44.9)	21 (42.9)	10 (50.0)
**Female**	66 (54.5)	38 (55.1)	28 (57.1)	10 (50.0)
**Age**		**Mean (SD)**	**Mean (SD)**	**Mean (SD)**
**Whole Sample**	59.1 (14.1)	57.5 (13.6)	58.7 (14.1)	54.5 (12.2)
**Female**	56.8 (13.7)	55.9 (12.5)	55.5 (13.4)	56.7 (10.5)
**Male**	62.9 (14.2)	59.5 (14.9)	62.8 (14.3)	52.4 (14.2)
**Location of Primary Melanoma**		**n(%)**	**n (%)**	**n (%)**
**Head and Neck**	22 (18.2)	11 (15.9)	8 (16.3)	3 (15.0)
**Lower Limbs**	32 (26.4)	19 (27.5)	13 (26.5)	6 (30.0)
**Upper Body**	46 (38.0)	25 (36.2)	19 (36.8)	6 (30.0)
**Upper Limbs**	21 (17.4)	14 20.3))	9 (18.4))	5 (25.0)
**Deprivation Quintile**		**n (%)**	**n (%)**	**n (%)**
**1 – Most Deprived**	2 (1.7)	0 (0)	0 (0)	0 (0)
**2**	4 (3.3)	1 (1)	1 (2)	0 (0)
**3**	21 (17.4)	16 (23.2)	11 (22.5)	5 (25.0)
**4**	38 (31.4)	25 (36.2)	18 (36.9)	7 (35.0)
**5 – Least Deprived**	56 (46.3)	27 (39.1)	18 (38.7)	8 (40.0)
**Rurality**			**N (%)**	
**Urban**	72 (59.5)	32 (46.4)	22 (44.9)	10 (50.0)
**Rural**	49 (40.5)	37 (53.6)	27 (55.1)	10 (50.0)

### How often were concerns submitted?

During the 12 months follow up, 61 participants reported active concerns about their skin using the ASICA app on 129 occasions [Median 2; Interquartile range (IQR) 1–2; Range (1–8)] Concerns were new pigmented skin lesions, changes to existing pigmented skin lesions, new or changing lumps associated with the skin, or a range of miscellaneous concerns including issues with their original primary melanoma excision scar, skin rashes, and nail changes (Table 2). The majority of reports comprised one or two concerns (109/129 (84%)), but on 14 occasions between three and eight separate concerns were included and detailed in the report (Table 2). Thus although 129 separate reports were submitted, they included a total of 190 separate skin concerns. The number of separate skin concerns submitted by any one individual throughout the trial varied from 1 to 16 [Median 2; IQR 1–3].

**Table 2:** Frequency and number of concerns* submitted using ASICA

**Number of reporting occasions per individual**	**Overall n (%)**	**Grampian n (%)**	**Cambridge n (%**)
**1**	28 (45.9)	19 (46.3)	9 (45)
**2**	20 (32.8)	14 (34.1)	6 (30)
**3**	8 (13.1)	4 (9.8)	4 (20)
**4**	1 (1.6)	1 (2.4)	0 (0)
**6**	2 (3.3)	2 (4.9)	0 (0)
**7**	1 (1.6)	0 (0)	1 (5)
**8**	1 (1.6)	1 (2.4)	0 (0)
**Test image only**	8	7	1
**TOTAL**	123 (100)	83 (100)	40 (100)
**Number of concerns included per report**	**Overall n (%)**	**Grampian n (%)**	**Cambridge n (%)**
**1**	86 (45.5)	61 (51.2)	26 (36.6)
**2**	23 (12.1)	17 (14.2)	6 (8.5)
**3**	7 (3.7)	4 (3.4)	3 (4.2)
**4**	4 (2.1)	1 (10.8)	3 (4.2)
**5**	0 (0)	0 (0)	0 (0)
**6**	2 (1.0)	0 (0)	2 (2.8)
**7**	0 (0)	0 (0)	0 (0)
**8**	1 (0.5)	1 (0.8)	0 (0)
**TOTAL**	189	119	71
**Number of concerns reported overall**			
**1**	21 (36.1)	13 (31.7)	9 (45.0)
**2**	19 (31.1)	15 (36.6)	4 (20.0)
**3**	6 (9.8)	6 (14.6)	0 (0)
**4**	4 (6.6)	2 (4.9)	2 (10.0)
**5**	2 (3.3)	1 (2.4)	1 (5.0)
**6**	2 (3.3)	0 (0.0)	2 (10.0)
**7**	0 (0)	0 (0)	0 (0)
**8**	1 (1.6)	1 (2.4)	0 (0)
**9**	1 (1.6)	1 (2.4)	0 (0)
**10**	0 (0)	0 (0)	0 (0)
**11**	2 (3.3)	1 (2.4)	1 (5.0)
**12**	0 (0)	0 (0)	0 (0)
**13**	0 (0)	0 (0)	0 (0)
**14**	0 (0)	0 (0)	0 (0)
**15**	1 (1.6)	0 (0)	1 (5.0)
**16**	1 (1.6)	1 (2.4)	1 (0)
**TOTAL**	**189**	**119**	**71**

*Concerns were new pigmented skin lesions, changes to existing pigmented skin lesions, new or changing lumps associated with the skin, or a range of miscellaneous concerns including issues with their original primary melanoma excision scar, skin rashes, and nail changes (see also Table 5)

### What was the range and nature of concerns submitted?

Table 3 summarizes the number of reports submitted and whether they related to the site of the first primary melanoma or another site. Approximately 62% (117/189) of reports submitted detailed concerns at sites other than the primary. Concerns about a new mole or changes to existing moles accounted for almost 60% of reports submitted. Further, 20% of concerns detailed “other concerns” such as skin rashes (2 diagnoses of shingles), nail changes and other types of non-pigmented skin lesions. Body location of reported concerns were roughly evenly distributed among head and neck, upper and lower limbs and torso, with a smaller number (6/189 (3.2%)) arising in the pelvic area.

**Table 3:** Nature of concerns indicated by reporting participant

	Overall	Grampian	Cambridge
	**n (%)**	**n (%)**	**n (%)**
**Number of reports submitted**	189 (100)	118 (100)	71 (100)
**Number of concerns raised relating to original primary site**	61 (31)	39 (33.1)	22 (31.0)
**Number of concerns relating to other site**	128 (69)	79 (66.9)	49 (69.0)
**Not clear**			
**Participant designation of concern**			
**New mole**	54 (28.6)	28 (23.7)	26 (36.6)
**Change in existing mole**	57 (30.2)	32 (27.1)	25 (32.2)
**Lump on skin**	28 (14.8)	24 (20.3)	4 (5.6)
**Lump under skin**	11 (5.8)	6 (5.1)	5 (7.0)
**Other**	39 (20.6)	28 (23.7)	11 (15.5)
**Location of concern reported**	**n (%)**	**n (%)**	**n (%)**
**Head and Neck**	33 (22.8)	25 (21.2)	18 (25.4)
**Upper Limbs**	43 (17.5)	23 (19.5)	10 (14.1)
**Lower Limbs**	47 (24.9)	27 (22.9)	20 (28.2)
**Torso**	60 (31.7)	37 (31.4)	23 (32.4)
**Pelvic Region**	6 (3.2)	6 (5.1)	0 (0)

### How good were the skin photographs that were submitted?

Almost a quarter (45/188 (23.9%)) of initially submitted skin photographs were of prime focused quality, with some blurring reported by the DNP in around two thirds (118/188 (62.7%)) (Table 4). Nevertheless 79 (41.4%) skin photographs were of sufficient quality to make a clinical decision. Further skin photographs were requested by the DNP on 111 occasions (58.6%). The patient returned these on 48 occasions (25.5%) but did not on 61 (33.5%) occasions. The rate of not returning further skin photographs appeared higher (53.5% vs 21.2%) in the Cambridge patients.

**Table 4:** Quality of submitted images, further images and clinical decisions

Quality of initial submitted images	Overall	Grampian	Cambridge
	**n (%)**	**n (%)**	**n (%)**
**Focused**	45 (23.9)	34 (28.8)	11 (15.5)
**Blurred**	118 (62.7)	67 (56.8)	51 (71.8)
**Not Stated**	25 (13.2)	16 (13.6)	9 (12.7)
**No Image Submitted**	1 (0.5)	1 (0.8)	0 (0)
	189	118	71
**Request for further images**			
**None requested**	79 (41.4)	61 (50.8)	18 (25.4)
**Requested and received**	48 (25.5)	33 (28.0)	15 (21.1)
**Requested and not received**	61 (33.5)	23 (21.2)	38 (53.5)
**Resolution of concern**	**n (%)**	**n (%)**	**n (%)**
**Resolved by assessment of ASICA images**	90 (47.8)	60 (51.3)	30 (42.3)
**Required further face-to-face assessment**	33 (17.5)	27 (23.1)	6 (8.5)
**Partial non-compliance by patient**	65 (34.6)	30 (25.6)	35 (49.3)
**Resolved with initial images**	62 (33.0)	42 (35.9)	20 (28.2)
**Resolved after further images sent**	28 (14.9)	18 (15.3)	10 (14.1)
**Seen at a secondary care clinic**	19 (10.1)	15 (12.8)	4 (5.6)
**Referred to GP**	14 (7.4)	10 (8.5)	4 (5.6)
**Benign but confirmatory further images not sent by patient**	17 (9.0)	2 (1.7)	15 (21.1)
**Not completed due to patient not sending further images**	48 (25.5)	29 (24.8)	20 (28.2)
	188	117	71

### How were participant concerns resolved?

Of the 189 concerns reported 188 included a skin photograph. 62 (32.8%) concerns were resolved in telephone conversation between the participant and the DNP and based on the initial skin photographs sent. On 28 occasions (14.8%) the concern was resolved when the participant and the DNP spoke initially and then again after the participant had submitted further skin photographs for assessment by the DNP. Face-to-face consultations were triggered on 33 (17.5%) occasions:: on 14 (7.4%) occasions participants were referred to their GP and for 19 (10.1%) participants an appointment was arranged at a face-to-face dermatology clinic. In around one third (66/189) of reports, the participants who reported a concern did not respond to a subsequent request for further images, with the DNP having made an assessment that the issue was initially benign for 17 of these. However, in total, this means that 49 concerns were not resolved by the DNP within the trial. It is important to be quite clear, however, that this had been anticipated in design and that the ASICA intervention was being delivered to participants in addition to their usual follow-up and primary and secondary care.

The range of assessments made by the DNP on participant reports of concern are shown in Table 5. The 61 “Benign (non-specific)” assessments were occasions where the DNP had felt that the patient could be reassured on the basis of his review of the clinical history and skin photographs submitted and did not require more detailed assessment or treatment. That there are a relatively large number of concern outcomes classified in this way, i.e. not attached to a specific diagnosis results from the fact that the trial clinician portal for receiving and detailing the assessment of submitted reports was improved about midway through the study enabling more clinical detail to be collected.

**Table 5:** Frequency of presumptive diagnoses made by Dermatology Nurse Practitioner

Diagnosis	N (%)
**Angioma**	3 (1.6)
**Angioma/Seborrhoeic Keratosis**	1 (0.5)
**Benign - Excoriated Papule**	1 (0.5)
**Benign - Insect Bite**	1 (0.5)
**Benign - Melanocytic Naevus**	1 (0.5)
**Benign - Trauma**	1 (0.5)
**Benign (non-specific)**	61 (32.3)
**Benign Naevus**	6 (3.2)
**Benign Naevus/Giant Comedone**	2 (1.1)
**Benign Naevus/Seborrhoeic Wart**	1 (0.5)
**Benign Nail Change**	1 (0.5)
**Benign Papilloma**	1 (0.5)
**Benign Papule (resolved)**	1 (0.5)
**Benign wart**	3 (1.6)
**Blister**	1 (0.5)
**Campbell De Morgan spot**	1 (0.5)
**Change in WLE scar (proved benign)**	1 (0.5)
**Concerning (Clinical Input Needed)**	1 (0.5)
**Concerning (non-specific)**	1 (0.5)
**Concerning Naevus**	2 (1.1)
**Concerning Naevus Between Toes**	1 (0.5)
**Dermal Naevus**	2 (1.1)
**Dermatitis**	2 (1.1)
**Dermatofibroma**	1 (0.5)
**Dry skin**	5 (2.6)
**Dry Skin (Chronic Venous Insufficiency)**	2 (1.1)
**Fungal Nail Infection (Not Relevant)**	1 (0.5)
**Local Inflammation**	1 (0.5)
**Meibomian Cyst**	1 (0.5)
**Nail Pigmentation**	1 (0.5)
**Nail Pigmentation (Traumatic)**	1 (0.5)
**None Formed**	49 (25.9)
**None Formed (had biopsy at clinic)**	1 (0.5)
**None Formed (resolved)**	2 (1.1)
**Not Relevant to Study**	2 (1.1)
**Paryonychia**	1 (0.5)
**Ruptured Hair Follicle**	1 (0.5)
**Seborrhoeic Keratosis**	19 (10.1)
**Shingles**	2 (1.1)
**WLE Scar**	1 (0.5)
**WLE Scar (healing)**	1 (0.5)
**WLE Scar foreign body, infection, recurrence**	1 (0.5)
**Total**	189 (100)

It was observed that individuals varied considerably in the interval between training and the first report of a concern being submitted. The first report was received within one month from 29 (42.0%) participants. The first report was received between one and three months by 20 (29.0%) participants, between three and six months from 11 (15.9%) participants and between six and 12 months from a further 9 (13.0%) participants. This demonstrates that the timing of individuals submitting their first report was spread throughout the study period and not clustered around training dates.

### What significant clinical events occurred?

These data are included to demonstrate practical applications of the intervention in a real-world setting. Table 6 details significant clinical events (diagnoses of metastatic melanoma, new primary melanoma and dysplastic naevi) and episodes where an ASICA report led to the DNP referring a patient to their GP or arranging for them to be seen face-to-face by a skin specialist in a secondary care clinic. As a result of using ASICA, 14 participants were referred to their GP and 19 face-to-face assessments were arranged at a dermatology outpatient clinic. We are not able to report on the detailed outcome of all these primary and secondary care encounters since we did not have sufficient access to primary and secondary care case-notes to triangulate these completely. Furthermore, due to the timescales involved not all episodes were concluded within the trial follow up period, with some appointments, results and procedures outstanding. We also collected data on diagnoses of recurrent melanoma and metastatic melanoma, new primary melanoma and new dysplastic lesions in the 12-month clinic reviews, since these are potential outcomes in a definitive trial of ASICA. Whilst we were able to collect this data, these events were rare with only two ASICA participants being diagnosed with metastatic melanoma, one with a new primary melanoma and one with two dysplastic naevi. These four events are detailed in Table 6 although, all occurred in the period between randomization and patients being trained and receiving the intervention. Thus, ASICA was not directly involved in any of these diagnoses. Table 6 also includes details of 5 of the 19 cases where patients were brought to a secondary care clinic for face to face assessment, where and details of the clinical outcome was available when outcome data were collected from participants’ medical records. In the further 14 cases details of the final clinical outcome were still awaited at the time outcome data were collected.

**Table 6:** Significant events and episodes of clinic or GP referral

Participant	Number of concerns	Significant event or GP/Secondary Care Clinic Referral	Clinical abstract	Definitive outcome if available
1	NA	Significant event	Randomized but metastases diagnosed subsequently. Underwent training but subsequently deceased.	Metastatic melanoma – deceased
2	3	Referred to GP	Submitted images of three naevi with benign appearance. Advised to see GP for further follow-up. No GP referrals made	
3	2	Clinic	Submitted images of two lesions on back. Underwent excision of these.	Not available
4	NA	Significant event	Diagnosed with dysplastic naevi between randomization and training. Underwent training on, used app during trial	New dysplastic naevus
5	1	Clinic	Submitted images of pigmented lesion near primary scar on right foot on. Subsequently underwent punch biopsy at Dermatology OPD on and attended for dressings. Benign compound naevus diagnosed	Benign naevus
6	1	Clinic	Sent images of itchy and raised areas on primary scar on right foot. Was seen by Consultant and DNP at clinic with no abnormality detected.	Not available
7	2	Clinic	Submitted images of two lesion on vertex of scalp. Had punch biopsies performed at plastic surgery.	Not available
8	1	Clinic	Submitted images of a pink lump around initial primary scar site. Was seen at clinic by Consultant and DNP including dermoscopy with no further concerns indicated.	Benign skin change
9	1	Clinic	Submitted image of lesion on right thigh. Seen at Plastic Surgery OPD for punch biopsy. Pathology reported benign dermatofibroma	Benign dermatofibroma
10	1	Clinic	Submitted images of new growth on primary scar on left thigh. Was seen at Dermatology OPD and found to have stitch within scar.	Foreign body in primary scar
11	2	Clinic	Submitted images of two lesion on back. Seen in Plastic Surgery OPD for excision biopsy of both.	Not available
12	2	Referred to GP	Submitted images on lesion of left arm on and discoloration under nail of left index finger. From initial and further images and history impression was of trauma to finger and benign papilloma. Referred to GP for further assessment if changing.	Benign papilloma and subungual haematoma
13	NA	Significant event	At time of randomization had pathology outstanding which proved to be a second primary. The participant was trained and continued in the trial.	Second primary melanoma diagnosed between recruitment and training. Participant continued to engage with trial.
14	1	Clinic	Submitted image of lesion on right eyebrow. Seen by DNP at Dermatology OPD diagnosed with seborrheic keratosis	Seborrheic keratosis
15	NA	Significant event	Randomized and trained – primary site on torso. Skin biopsy before randomization proved to be a metastatic deposit on left shin. Participant continued in the trial. Submitted images of new skin rash and seen in Dermatology OPD. Diagnosis was drug induced rash secondary to Pembrolizumub.	Metastatic melanoma
16	1	Clinic	Submitted image of possible changes in existing mole on right leg. Seen at Dermatology clinic (date not available) diagnosis was benign, no procedure recorded.	Not available
17	1	Referred to GP	Submitted image of new naevus on right lower abdomen. Had been seen by earlier by Plastic Surgeon not concerned. DNP made benign assessment based on images asking patient to consult with GP if further changes.	Not available
18	1	Clinic	Submitted image of warty lesion on left ankle. Was seen in Plastics OPD and excision biopsy on. Subsequent pathology revealed a seborrheic wart.	Seborrheic wart
19	1	Clinic	Submitted image of lesion on left cheek/pre-auricular area. Was seen in Dermatology clinic by DNP. Lesion subsequently excised by Plastic Surgeon with subsequently pathology reporting a basal cell carcinoma	Basal cell carcinoma
20	1	Referred to GP	Submitted images of “erythematous papule with some telangectasia” on left upper back. Asked to consult GP for further assessment. GP referred patient to Dermatology and was seen first and then again for punch biopsy. Pathology reported a ruptured hair follicle.	Ruptured hair follicle
21	3	Referred to GP	Participant submitted blurred images of three potentially new lesions. DNP called and elicited no worrying features in history. Invited new images or suggested GP review as easier for older rural patient. Patient saw GP, no worrying features and no further referral deemed necessary.	Not available
22	2	Referred to GP	Participant submitted images of two warty lesion on left forearm. Further images requested by DNP revealing no worrying features in history or appearance. Participant asked to consult GP if changes with no subsequent GP referral.	Not available
23	1	Clinic	Submitted image of lesion on mid-back. Referred to Dermatology clinic and seen. Subsequent pathology reported as “Dysplastic naevus.”	Dysplastic naevus
24	1	Clinic	Submitted image of lesion on right upper abdomen “existing mole become scaly.” Referred to Dermatology clinic and seen. Details of outcome not available.	
25	1	Referred to GP	Submitted images of lump in lower mid-lumbar area. Contacted by DNP and advised to see GP for assessment. DNP contacted in one week GP had diagnosed “lipoma” and referred to Dermatology outpatient clinic. See there with subsequent diagnosis of “spindle cell lipoma.” Not clear if biopsy was performed.	Spindle cell lipoma
26	1	Clinic	Patient submitted image of new red area around scar site feeling slight raised and blanching with pressure. Was referred to Dermatology clinic and had appointment within two weeks. No further details of outcome available.	Not available
27	1	Referred to GP	Submitted images of new lesion on anterior right thigh. Following further images and phone call with DNP decided lesion was benign. But participant advised to monitor and report changes to GP. No subsequent GP referrals noted.	Not available
28	1	Clinic	Submitted image of discolouration in nailbed of right thumb under nail. Referred to Dermatology clinic and seen by consultant. Given reassurance and no further action required.	Not available

### Practical experiences of users and the dermatology nurse practitioner?

Telephone interviews were conducted 13 members of the intervention group. Of these eight were male, five were female and the age range was 39–86. Eight lived in a rural setting and five in an urban setting. Four were from the Cambridge site and nine from Grampian. The interviews demonstrated a range of experiences, behaviours, beliefs and feelings with respect to their skin, skin checking and technology. Most participants stated that they had already checked their skin regularly, but that using ASICA increased frequency/consistency of checking and supported a more systematic approach to skin-checking. Some users also reported that ASICA had improved their confidence about when and how to check their skin. ASICA users were almost universally well-disposed to technology becoming integral part of their health care, with the caveat that personal interaction DNP had helped make the intervention work. Most saw the potential of digital technology and the likelihood that it will be increasingly used to facilitate healthcare in the future. Participants and the study DNP made several suggestions to improve usability and functionality. The hardware provided, was criticised, with several participants reporting issues with the camera and operating system. The hardware (i.e. the tablet) was reported as the main barrier to the ASICA app’s use. Most participants did not believe ASICA had changed their feelings around skin checking, but it had raisied awareness and changed their own skin checking behaviours for the better. Overall, using ASICA did not appear to increase anxiety in the long-term. Some participants suggested that ASICA temporarily and briefly increased anxiety about skin-checking at the start, but that this has settled over time with regular use. In contrast, several participants viewed the app as a means of accessing rapid reassurance when concerns arose.

## Discussion

### Summary of main findings

This feasibility RCT has demonstrated that over half of the participants previously treated for melanoma randomized to the intervention group (57%) were able to use the ASICA intervention can enable to report concerns with their skin. Most of these (74.5%) then interacted with a remote specialist DNP to resolve these concerns. The participants who used ASICA were demographically diverse. Further, it appeared that those reporting concerns, compared to the intervention group overall, were slightly younger, more likely to live rurally and less likely to be socioeconomically deprived. There was also a greater tendency for those at the Cambridge site to not send a further skin photograph when requested by the DNP. Participants use of ASICA to report skin concerns appeared to be sustained throughout the 12-month study period as opposed to concentrated around training with subsequent decline, with a wide-range of different skin concerns reported. The effectiveness of the intervention appeared to be constrained by the quality of the images achieved with the device and also by participants frequently defaulting from sending further images. A small number of significant events occurred in the intervention group, but their detection was did not result from ASICA use. On the other-hand the trial demonstrated the facility of the ASICA intervention to recognize significant concerns which could be referred to participants’ GPs or hospital out-patient departments for subsequent resolution.

### Context with other literature

Experience of using digital healthcare generally, and for melanoma follow-up in particular, is growing but good quality evidence from rigorous real-world trials is required to move interventions from the innovation and introductory phase to become an effective means of healthcare delivery [28]. In that context the data presented here provide useful evidence of the scope and potential of a sequentially developed intervention to effectively support remote follow-up for those treated for melanoma. The data presented here are useful in providing reassurance that the common concerns of those in melanoma follow-up can be appropriately identified using remote technology. This accords with the findings of literature review which reviewed 114 papers (including 14 systematic reviews) capturing 20 years of tele-dermatology research and concluding that it is an efficient and effective healthcare service compared to in-person care [29]. The reviewers further concluded that tele-dermatology reduces patients’ travel time and waiting time, avoids (unnecessary) dermatologic visits, and improves access of care to underserved patients. Our data echoes this to some extent, since over half of our participants using the intervention successfully were rural residents, and they also appeared slightly more likely to do so than the urban recruits [29].

On the other hand, those from a deprived background were under-represented in our study, as in many other studies of digital healthcare, which represents a challenge for future researchers. Further, of six recruits from the two most deprived quintiles, only one reported a concern using ASICA in the year. This accords with a further review of using smartphones and instant messaging which found evidence of their potential to support remote dermatology in the developing world, so it seems self-evident that there is a need for research to understand how socioeconomically disadvantaged populations in the developed world can benefit most [30]. On the other hand the fact that six individuals from the UKs most deprived quintiles have participated in this trial, and at least one individual used the intervention successfully is encouraging, but suggest that much more must be done to engage those of lower socioeconomic status in developing digital healthcare. A further world-wide review emphasized the growing scope and potential of tele-dermatology to deliver many different aspects of remote dermatology care [31]. The review concluded that teledermatology increases patient satisfaction, reduces wait times and decrease costs. Underserved communities and those in rural settings are also more likely to have a dermatologic evaluation by a specialist via teledermatology [31]. Our data certainly provide objective evidence to support these points. One caveat, however, is our finding that those at the Cambridge site were considerably less likely to submit second skin photographs when requested by the DNP, compared to those individuals in the same health board area as him. This could be because that individual was more relatable to individuals in the same area, which has implications for future upscaling of digital healthcare delivery. Finally, our data provide important information about how participants have interacted with a digital healthcare intervention, which strongly supports a view of digital interventions as a means for “testing and advancing theories of behaviour change by generating ecologically valid, real-time objective data.” [32] This point will be developed in a further paper reporting on adherence in the ASICA trial.

### Strengths and limitations

ASICA was a relatively large two-centred feasibility trial of a novel digital intervention to prompt, record and respond remotely to total-skin-self-examination by those previously treated for melanoma. Consequently, this approach confers the advantage that the approach and results reported here were rigorously produced. We have demonstrated that patients treated for melanoma are willing to be recruited to a trial of remote and self-directed follow-up and that it appears attractive to people across the demographic range of those diagnosed with melanoma. We have also demonstrated that the target group were willing to be randomized, which is useful information for a subsequent definitive evaluation. The current report further demonstrates that ASICA is sufficiently technologically robust to enable a remote DNP in Northeast Scotland to complete rapid and appropriate assessment of concerns submitted by participants at sites in both Scotland and England. Further we have learned much about how the intervention interface needs to be improved and reprogrammed for subsequent development, evaluation and eventual implementation. We have also gained much knowledge about trial processes for further definitive evaluation.

We acknowledge several limitations. With advances in technology, some aspects of the ASICA intervention proved unwieldy in the pilot trial. As the presented data demonstrate, the initial images submitted by patients were frequently of insufficient quality for the DNP to make a reasonable initial assessment. This resulted in participants needing to be contacted to submit further images, introducing delay which was antithetical to the purpose of the intervention. This led to the most striking limitation of the study which was that a considerable number of participants did not submit further photographs when requested, meaning that the initial concern raised could not be completely resolved. Unfortunately, we do not have the data to explain this sufficiently. However, we would speculate that, since most of the concerns submitted during the trial were benign in nature, on the occasions when participants defaulted on submitting further photographs may be because their level of concern was not high enough to motivate the further effort of taking and submitting further images. It should also be pointed out that all the participants were still in receipt of regular structured hospital follow-up and may have been sufficiently close to their next appointment to await that when a technical challenge arose. We believe, however, that once the technical specification of ASICA can be improved this issue will be mitigated. A further potential limitation is under-reporting of issues on hard-to-see areas of the body and it is notable, for example, how few concerns from the pelvic region were submitted. An additional limitation to the further development of ASCIA is that the DNP receiving and acting on the reports is a highly skilled and experienced DNP comfortable with remotely assessing concerns submitted by this high-risk group. If ASICA is to be used at scale in future, appropriately skilled individuals will need to be identified or training will need to be developed for less experienced individuals. We were reassured that most of the concerns submitted during the present trial were non-concerning, with only a relatively small number triggering GP or hospital appointments. Consequently, we believe it will be possible to train less-specialized nurses to undertake initial triage of submitted lesions and to recognize many of the concerns submitted. It should also be noted that the trial was conducted over 12 months only and subsequent evaluation should explore whether use of ASICA to support TSSE can become sustained in the medium to longer-term. It must also be acknowledged that a considerable proportion of intervention group participants did not use ASICA and, at the time of writing, a detailed analysis of ASICA adoption and adherence is being completed and will subsequently be submitted for publication. Additionally, a proportion of users and non-users took part in qualitative interviews about their experiences. We have reported data from the ASICA users here but a fuller report which will also be published separately.

## Conclusion and implications

The ASICA feasibility RCT has demonstrated that most skin concerns submitted by melanoma survivors, and ranging from minor skin rashes to new moles, can be assessed remotely. ASICA can also facilitate prompt referral to GPs and secondary care clinics for concerning cases where appropriate.. Overall, there is good evidence that, with appropriate future development, ASICA has the potential to transform melanoma survivorship care.

## Data Availability

The datasets generated and/or analysed during the current study are not publicly available due to concerns about the potential for individual participants to be identified from the data and due to the scope of the ethical approvals received. However data may be available from the corresponding author on reasonable request and subject to appropriate safeguards.
